# Use of Biomarkers of Inflammation in the Differentiation of Iron Deficiency and Anaemia—Lessons from Inflammatory Bowel Disease

**DOI:** 10.3390/diagnostics14141515

**Published:** 2024-07-13

**Authors:** Karima Farrag, Aysegül Aksan, Valëza Ademaj-Kospiri, Eleni Leventi, Jürgen Stein

**Affiliations:** 1 Innere Medizin, DGD Kliniken Sachsenhausen, Schulstrasse 31, 60594 Frankfurt am Main, Germany; j.stein@em.uni-frankfurt.de; 2 Interdisciplinary Crohn Colitis Centre Rhein-Main, Schifferstr. 59, 60594 Frankfurt am Main, Germany; ayseguel.aksan@ernaehrung.uni-giessen.de (A.A.); valeza.ademaj@gmail.com (V.A.-K.); lena.leventi@gmail.com (E.L.); 3 Institute of Nutritional Science, Justus Liebig University, 35392 Giessen, Germany; 4 Radiologie, Klinikum Aschaffenburg-Alzenau, Am Hasenkopf 1, 63739 Aschaffenburg, Germany

**Keywords:** inflammatory bowel disease (IBD), ferritin, C-reactive protein (CRP), α1-acid glycoprotein (AGP), iron deficiency

## Abstract

Iron deficiency and iron deficiency anaemia are common in inflammatory bowel disease (IBD), to the detriment of the patients’ quality of life. Since ferritin, as an acute-phase protein (APP), has limited diagnostic value in IBD, concurrent assessment of C-reactive protein (CRP) is recommended. The World Health Organization suggests using α1-acid glycoprotein (AGP) as an additional biomarker due to its differing half-life. This study aimed to evaluate ferritin levels in patients with IBD using CRP and AGP, individually and in combination. A total of 118 patients with IBD (mean age: 45.48 ± 15.25 years, 47.46% female) were recruited, including 38 with Crohn’s disease, 47 with ulcerative colitis, and 33 controls. The results showed that while CRP alone detected an inflammatory increase in ferritin of 29.76%, this increased to 82.14% when AGP or both AGP and CRP were considered (*p* < 0.05). Elevated AGP levels were more prevalent in patients with ulcerative colitis. However, concordance between high CRP and AGP levels was confirmed in only 55% of cases. Correcting for inflammation using CRP and/or AGP significantly improved the diagnostic accuracy of ferritin levels in patients with IBD, highlighting the challenge posed by inflammation when assessing iron deficiency.

## 1. Introduction

Iron deficiency (ID) is a common extra-intestinal manifestation of inflammatory bowel disease (IBD) that significantly affects the daily functioning and overall health of individuals, regardless of the presence of anaemia [1,2,3,4]. A comprehensive understanding of the underlying mechanisms of ID and its association with different types of anaemia is crucial for the development of effective diagnostic markers and therapeutic strategies [5,6,7].

Depending on the underlying pathophysiology, two distinct types of ID can be distinguished, each presenting with identical clinical symptoms: (1) absolute ID (AID), characterised by a quantitative decrease in iron stores due to factors such as inadequate dietary iron intake, iron malabsorption, or chronic blood loss from the gastrointestinal tract, which ultimately leads to iron deficiency anaemia (IDA) if left untreated [8] and (2) functional ID (FID), which results from the sequestration of iron in patients with otherwise normal or abundant stores, leading to the development of anaemia of chronic disease (ACD) if left untreated [9,10]. Importantly, these two types of ID often coexist and are not mutually exclusive.

In clinical practice, serum ferritin and transferrin saturation (TSAT) are routinely considered the most effective biomarkers of iron status, with guidelines widely endorsing their use for initial assessment [11,12,13,14]. However, despite its utility as an indicator of stored iron, ferritin is also an acute-phase protein (APP) whose synthesis can be upregulated by cytokines during inflammation, independent of iron homeostasis [15,16]. Although investigations have explored the utility of higher ferritin cut-off values during acute-phase responses such as infection or inflammation, these findings lack robust evidence-based support [17]. Consequently, there is a need to identify markers that can factor in inflammation when interpreting iron deficiency, particularly in patients with inflammatory bowel disease (IBD), in whom chronic inflammation persists. The Working Group of the World Health Organization (WHO) thus recommends the simultaneous measurement of ferritin and two other APPs, namely C-reactive protein (CRP) and alpha-1-acid glycoprotein (AGP), to determine the presence of an acute-phase response [15,18].

The rationale for measuring both CRP and AGP lies in their different response patterns during an acute-phase reaction [19]: CRP shows a rapid rise in response to inflammation, peaking within 24–48 h, whereas AGP may take several days to reach peak levels after the onset of inflammation. Therefore, the inclusion of AGP is particularly advantageous in the detection of chronic or subclinical inflammation [20,21]. Erythrocyte sedimentation rate (ESR), with a half-life of approximately 19 h, is another valuable marker of inflammation, showing an early rise within 12–24 h of inflammation onset and peaking in 2–3 days. Thus, ESR serves as a useful adjunct in the detection of inflammation [21,22].

Our study aims to elucidate the relationship between established biomarkers of inflammation (ESR, CRP, and AGP) and their associations with markers of iron status. Specifically, we investigated whether these biomarkers, either individually or in combination, improve diagnostic accuracy for ID in patients with IBD-related inflammation.

## 2. Materials and Methods

This study was designed as a comparative, cross-sectional, and retrospective investigation involving adult patients previously diagnosed with IBD. Participants consecutively attended follow-up consultations as outpatients at the Interdisciplinary Crohn’s and Colitis Centre (ICCC) Rhein-Main, located in Frankfurt am Main, Germany.

Ethical considerations were paramount throughout the study process. The research adhered strictly to the ethical principles outlined in the Declaration of Helsinki. Approval for the study protocol was obtained from the local ethics committee, the Landesärztekammer Hessen, under the reference number 2019-1317-evBO.

### 2.1. Population Characteristics

This study included data from eligible patients aged 18–65 years who consecutively attended the clinic and had previously been diagnosed with IBD on the basis of standard clinical, radiological, and pathological criteria [23].

Exclusion criteria for this study were incomplete medical records, cytopoenia induced by oral or intravenous azathioprine, liver failure, recent blood donation, blood loss, iron supplementation, or plasmapheresis within the last three months prior to laboratory testing. All potentially eligible patients were individually informed about all aspects of this study, including procedures and data protection measures, and written informed consent was obtained before their data were collected for inclusion in the analysis.

### 2.2. Study Design

Demographic data, disease characteristics, details of ongoing treatment, and laboratory results, including inflammation and iron markers, were obtained and documented from electronic patient records. Blood samples were collected between 08:00 and 10:00 h during routine follow-up visits and stored at −30 °C until analysis.

Concentrations of the relevant parameters were analysed from serum samples according to the guidelines of the German United Society for Clinical Chemistry and Laboratory Medicine (Deutsche Vereinte Gesellschaft für Klinische Chemie und Laboratoriumsmedizin, DGKL) at a local reference laboratory (Laborarztpraxis Dres. med. Walther, Weindel und Kollegen MVZ GbR, Frankfurt am Main, Germany) using a standard methodology. Disease localisation of IBD was defined according to the Montreal Classification [24].

### 2.3. Inflammatory Markers

In this study, we evaluated the diagnostic utility of various inflammatory markers, including AGP, high-sensitivity CRP (hsCRP), and ESR, both individually and in combination. Inflammation was defined by elevated levels of AGP (≥1000 µg/mL) [25], hsCRP (≥5 mg/dL) [26], and/or ESR (≥15 mm/h for males and ≥20 mm/h for females aged 18–50 years and ≥20 mm/h for males and ≥30 mm/h for females aged 51–65 years) [27]. All patients with at least one elevated inflammatory marker (AGP, CRP, or ESR) according to these limits were included in the inflammation group.

### 2.4. Definition of Iron Deficiency and Anaemia

Iron deficiency was defined as AID when serum ferritin was <30 μg/L and TSAT < 20%. FID was defined as ferritin levels between 30 and 100 μg/L with TSAT < 20% in the presence of inflammation [28].

Anaemia was defined as a haemoglobin (Hb) concentration below 13 g/dL for males and 12 g/dL for females according to the WHO criteria [18]. The types of anaemia were subclassified as follows: IDA when TSAT was <20% and ferritin < 30 μg/L in the absence of inflammation; ACD when TSAT was <20% and ferritin ≥ 100 μg/L in the presence of inflammation; and mixed anaemia (MIX) when TSAT was <20% and ferritin was between 30 and 100 μg/L in the presence of inflammation [29,30].

### 2.5. Statistical Analysis

The data were analysed using IBM SPSS version 25.0 (IBM Corporation, Armonk, NY, USA) and Microsoft Office Excel 365 (Microsoft Corporation, Redmond, WA, USA). The normal distribution of variables was assessed using the Kolmogorov–Smirnov test. Descriptive statistics were reported as mean ± standard deviation for normally distributed variables and median with minimum–maximum values for variables that were not normally distributed. Categorical variables were presented as frequency and percentages.

Parametric or non-parametric tests were selected based on the statistical characteristics of the data analysed and adherence to the assumptions of parametric tests. Receiver operating characteristic (ROC) curve analysis was employed to assess and compare the diagnostic performance of inflammatory markers. Statistical significance was defined as *p* < 0.05.

## 3. Results

### 3.1. Study Population

Table 1 shows the general characteristics of the study population stratified by disease type. A total of 170 patients with IBD were included, comprising 93 patients with Crohn’s disease (CD) and 77 patients with ulcerative colitis (UC). Ninety-three of the 170 patients (54.7%) were female. The mean age was 43.5 ± 13.1 years for patients with CD and 47.2 ± 13.0 years for those with UC. No statistically significant differences were observed between the CD and UC groups with respect to gender distribution, age, disease location, medication therapies, or baseline laboratory characteristics.

### 3.2. Iron and Inflammation Markers According to Inflammation Status

The laboratory characteristics of patients with and without inflammatory activity are described in Table 2. Mean Hb and TSAT levels were significantly higher in patients without active inflammation compared to the group of patients with inflammation (Hb: 14.2 (10.1–16.9) g/dL vs. 13.5 (7.0–16.3) g/dL and TSAT: 24.5 (3.9–38.3) % vs. 14.2 (2.8–36.6) %, respectively, for non-inflammatory and inflammatory disease states).

Serum ferritin and transferrin levels did not show significant differences between the inflammatory and non-inflammatory disease activity groups (ferritin: 67.7 (7.3–536.0) ng/mL vs. 85.6 (5.0–1180.0) ng/mL and transferrin: 266.00 (77.3–416.0) mg/dL vs. 257.0 (143.0–407.0) mg/dL, respectively, for non-inflammatory and inflammatory disease states).

In addition, all inflammatory markers, including ESR, hsCRP, and AGP, were significantly elevated in the group with inflammatory disease activity compared to the non-inflammatory group. The median ESR values were 117.0 mm/h (2.0–79.0) in the inflammatory group and 4.0 mm/h (1.0–25.0) in the non-inflammatory group (*p* < 0.001). The median hsCRP levels were 7.7 mg/L (1.0–112.0) in the inflammatory group and 1.2 mg/L (range 1.0–4.9) in the non-inflammatory group (*p* < 0.001). The median AGP concentrations were 976.0 µg/mL (469.7–1610.0) in the inflammatory group and 612.2 µg/mL (227.3–984.9) in the non-inflammatory group (*p* < 0.001).

### 3.3. Correlations of the Inflammatory Markers

**Figure 1:**
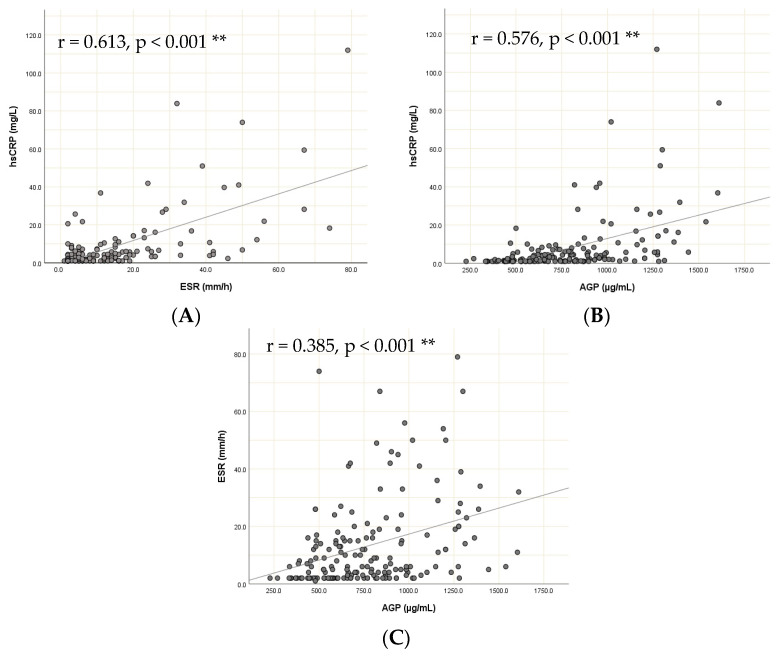
(**A**) Correlation of hsCRP with ESR (r = 0.613, *p* < 0.001 **); (**B**) correlation of hsCRP with AGP (r = 0.576, *p* < 0.001 **); and (**C**) correlation of ESR with AGP (r = 0.385, *p* < 0.001 **). ESR, erythrocyte sedimentation rate; hsCRP, high-sensitivity C-reactive protein; and AGP, α1-acid glycoprotein.

Figure 1 shows the correlations between the different markers of inflammation. hsCRP, AGP and ESR were all found to be significantly correlated.

The correlations between hsCRP and ESR (r = 0.613, *p* < 0.001) and hsCRP and AGP (r = 0.576, *p* < 0.001), respectively, were moderate, whereas that between ESR and AGP (r = 0.385, *p* < 0.001) was low.

### 3.4. Correlations of Serum Inflammatory Markers with Iron Parameters

Table 3 shows the correlations between the inflammatory markers and a selection of other analysed laboratory parameters. Ferritin exhibited a significant positive correlation with hsCRP (r = 0.180, *p* = 0.019) and ESR (r = 0.163, *p* = 0.033). The correlation between AGP and ferritin was positive but not statistically significant.

Similarly, Hb had significant negative correlations with hsCRP (r = −0.547, *p* < 0.001) and ESR (r = −0.194, *p* = 0.011). The correlation between AGP and Hb was negative but without statistical significance. Transferrin exhibited significant negative correlations with hsCRP (r = −0.192, *p* = 0.012) and AGP (r = −0.227, *p* = 0.003), while the correlation between ESR and transferrin was negative but not statistically significant.

TSAT showed significant negative correlations with all inflammatory markers: hsCRP (r = −0.538, *p* < 0.001), ESR (r = −0.531, *p* < 0.001), and AGP (r = −0.338, *p* = 0.001).

### 3.5. Analytical Performance of Inflammatory Markers for the Detection of Active Inflammation

**Figure 2:**
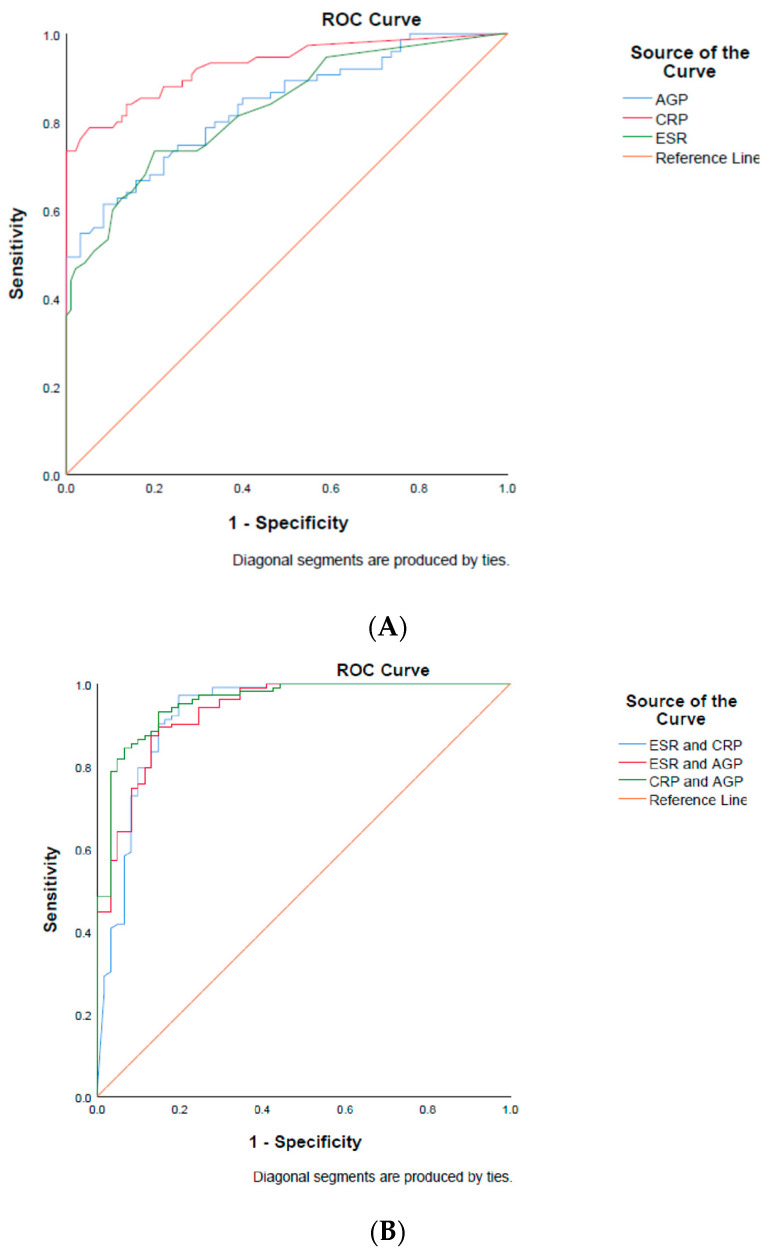
(**A**): Receiver operating characteristic (ROC) curves of the inflammatory markers AGP, CRP, and ESR alone and (**B**) receiver operating characteristic (ROC) curves of the combinations of the inflammatory markers AGP, CRP, and ESR.

ROC analysis was performed to compare different parameters as detectors of inflammation and to summarise the performance of each parameter as a sole marker and as a combination of two markers (Figure 2A,B and Table 4). As a sole biomarker, compared to ESR and AGP, hsCRP demonstrated superior diagnostic performance in the detection of inflammation (AUC values: 0.828, 0.846, and 0.928, respectively, for ESR, AG, and hsCRP). Overall, any combination of the inflammatory markers showed a better diagnostic performance for detecting inflammation than each parameter alone. The best of these combinations was AGP and hsCRP, with an AUC of 0.955, sensitivity of 93%, and specificity of 85%.

## 4. Discussion

Diagnosing ID in individuals with IBD poses a significant challenge, particularly when inflammation is present. This is the first observational study to evaluate α1-acid glycoprotein (AGP) as a third acute-phase protein marker to aid in diagnosing ID in patients with IBD. Typically, serum ferritin is recognized as the most accurate indicator of body iron stores in the absence of inflammation. However, during active inflammation, serum ferritin levels can be falsely normal or elevated due to its role as an acute-phase reactant, complicating the interpretation of iron status in these patients [23,31].

Several studies underscore the importance of measuring biomarkers of inflammation when assessing iron status. Research on infants and preschool children has suggested that predictors of inflammation vary by setting. This highlights the necessity of biochemical indicators for detecting subclinical inflammation [32,33].

Our data highlight the challenge of assessing ID and IDA using only serum ferritin as a marker of iron status in patients with IBD and active inflammation. Our study shows the importance of measuring both CRP and AGP concentrations: These markers reflect different phases of the acute-phase response, ranging from acute infection (rapid onset within one hour) to chronic inflammation (rising after 24 h and lasting four to five days) [19,34]. As the intensity of infection diminishes, CRP levels fall rapidly, whereas AGP remains elevated over a longer period. In contrast, ferritin rises quickly within a few hours of trauma and remains elevated even after CRP concentrations have subsided, while AGP concentrations are still increased [20].

Diagnosing true iron deficiency independent of inflammation is crucial for choosing the appropriate treatment approach and avoiding unnecessary treatment-related adverse events [35]. Thurnham et al. showed in their meta-analyses that the increase in ferritin levels was greater when CRP (50%), rather than AGP (38%), was elevated [21]. Consequently, they strongly recommend using a combination of CRP and AGP since together, they cover the full inflammation cycle, resulting in unbiased estimates of prevalence [36].

Our study found that the assumption that most patients with high CRP concentrations would also have high concentrations of AGP, and vice versa, was not supported; this was true in only 62.75% of cases. Thus, future studies on iron status in patients with IBD should carefully consider which APP biomarker is better at predicting elevated ferritin. Neither CRP, ferritin, nor AGP alone can adequately discriminate between individuals with low and high ferritin levels.

In conclusion, our study stresses the limitations of currently available APPs for predicting elevated ferritin levels based on inflammation. The percentage of patients with any one abnormal APP value was significantly higher than when CRP was used alone, indicating the necessity of a multi-marker panel when interpreting ferritin levels in the context of inflammation.

## Figures and Tables

**Table 1:** Patient characteristics according to disease type.

	Crohn’s Disease	Ulcerative Colitis	p_1_	p_2_
*N* (female)	93 (52)	77 (41)	0.759	-
Age (years), mean ± SD	43.5 ± 13.1	42.7 ± 13.0	-	0.869
Disease localisation				
Ulcerative colitis, *n* (%)				
E1 proctitis	-	4 (5.2)	-	-
E2 left-sided colitis		47 (70.0)		
E3 pancolitis		26 (33.8)		
Crohn’s disease, *n* (%)				
L1 ileum	43 (46.2)	-	-	-
L2 colon	19 (20.4)			
L3 ileocolic	26 (28.0)			
L4 perianal	5 (5.4)			
Medication, *n* (%)				
No treatment	5 (5.4)	6 (7.8)	0.548	-
5-ASA	16 (17.2)	22 (28.6)	0.096	-
Corticosteroids	6 (6.5)	8 (10.4)	0.404	-
Immunomodulators	5 (5.4)	1 (1.3)	0.223	-
Biologicals	62 (66.7)	41 (53.2)	0.084	-
Laboratory markers, median (min-max)				
Hb (g/dL)	13.9 (8.2–16.9)	13.9 (7.0–16.5)	-	0.820
Ferritin (ng/mL)	85.6 (6.2–1180.0)	55.2 (5.0–1166.0)	-	0.094
Transferrin (mg/dL)	254.0 (77.3–416.0)	272.0 (143.0–404.0)	-	0.138
TSAT (%)	17.8 (3.5–37.5)	20.1 (2.8–38.3)	-	0.852
ESR (mm/h)	6.0 (1.0–67.0)	7.0 (2.0–79.0)	-	0.846
hsCRP (mg/L)	3.8 (1–0-83.9)	2.0 (1.0–112.0)	-	0.068
AGP (µg/mL)	789.0 (335.8–1610.0)	709.7 (227.3–1386.9)	-	0.092

p_1_: chi-square test, p_2_: Mann–Whitney U test. Hb, haemoglobin; TSAT, transferrin saturation; ESR, erythrocyte sedimentation rate; hsCRP, high-sensitivity C-reactive protein; and AGP, α1-acid glycoprotein.

**Table 2:** Laboratory markers according to inflammation status.

	Inflammatory (*n* = 75) (ESR and/or hsCRP and/or AGP over Cut-Off ^§^)	Non-inflammatory (*n* = 95) (ESR and hsCRP and AGP under Cut-Off ^§^)	*p*
Hb (g/dL)	13.5 (7.0–16.3)	14.2 (10.1–16.9)	0.004 *
Ferritin (ng/mL)	85.6 (5.0–1180.0)	67.7 (7.3–536.0)	0.177
Transferrin (mg/dL)	257.0 (143.0–407.0)	266.00 (77.3–416.0)	0.162
TSAT (%)	14.2 (2.8–36.6)	24.5 (3.9–38.3)	<0.001 **
ESR (mm/h)	117.0 (2.0–79.0)	4.0 (1.0–25.0)	<0.001 **
hsCRP (mg/L)	7.7 (1.0–112.0)	1.2 (1.0–4.9)	<0.001 **
AGP (µg/mL)	976.0 (469.7–1610.0)	612.2 (227.3–984.9)	<0.001 **

* *p* < 0.05 and ** *p* < 0.001: Mann–Whitney U test, ^§^ ESR cut-off: for females and males under 50 years, 20 mm/hg and 15 mm/hg, respectively; for females and males 51 years and over, 10 mm/hg and 5 mm/hg, respectively; hsCRP cut-off: 5 mg/dL; and AGP cut-off: 1000 µg/mL. Hb, haemoglobin; TSAT, transferrin saturation; ESR, erythrocyte sedimentation rate; hsCRP, high-sensitivity C-reactive protein; and AGP, α1-acid glycoprotein.

**Table 3:** Correlations of serum inflammatory markers with iron parameters in patients with IBD.

		Spearman’s Rho	*p*
ESR (mm/h) vs	Haemoglobin [g/dL]	−0.547	<0.001 **
	Ferritin (ng/mL)	0.163	0.033 *
	Transferrin (mg/dL)	−0.046	0.552
	TSAT (%)	−0.531	<0.001 **
hsCRP (mg/L) vs	Haemoglobin [g/dL]	−0.194	0.011 *
	Ferritin (ng/mL)	0.180	0.019 *
	Transferrin (mg/dL)	−0.192	0.012 *
	TSAT (%)	−0.538	<0.001 **
AGP (µg/mL) vs	Haemoglobin [g/dL]	−0.097	0.209
	Ferritin (ng/mL)	0.140	0.069
	Transferrin (mg/dL)	−0.227	0.003 *
	TSAT (%)	−0.338	<0.001 **

* *p* < 0.05 and ** *p* < 0.001. TSAT, transferrin saturation; ESR, erythrocyte sedimentation rate; hsCRP, high-sensitivity C-reactive protein; and AGP, α1-acid glycoprotein.

**Table 4:** Analytical performance of inflammatory parameters to detect the presence of inflammation.

	AUC^ROC^	Sensitivity	Specificity	*p*
ESR	0.828 (0.766–0.891)	60.0%	89.5%	<0.001 **
hsCRP	0.928 (0.887–0.969)	72.0%	100.0%	<0.001 **
AGP	0.846 (0.781–0.910)	62.0%	92.0%	<0.001 **
ESR and hsCRP	0.932 (0.888–0.976)	97.0%	82.0%	<0.001 **
ESR and AGP	0.934 (0.897–0.972)	89.0%	85.0%	<0.001 **
AGP and hsCRP	0.955 (0.926–0.985)	93.0%	85.0%	<0.001 **

** *p* < 0.001. ESR cut-off: for females and males under 50 years, 20 mm/hg and 15 mm/hg, respectively; for females and males of 51 years and over, 10 mm/hg and 5 mm/hg, respectively; hsCRP cut-off: 5 mg/dL; AGP cut-off: 1000 µg/mL. AUC^ROC^, area under curve receiver operating characteristic; ESR, erythrocyte sedimentation rate; hsCRP, high-sensitivity C-reactive protein; and AGP, α1-acid glycoprotein.

## Data Availability

The data underlying this article are available in the article and can be obtained from the authors upon reasonable request.

## Abbreviations

ACD	anaemia of chronic inflammation
AGP	α1-acid glycoprotein
AID	absolute iron deficiency
APP	acute-phase protein
AUC	area under curve
CD	Crohn’s disease
CRP	C-reactive protein
ESR	erythrocyte sedimentation rate
FID	functional iron deficiency
Hb	haemoglobin
hsCRP	high-sensitivity C-reactive protein
IBD	inflammatory bowel disease
ID	iron deficiency
IDA	iron deficiency anaemia
ROC	receiver operating characteristic
TSAT	transferrin saturation
UC	ulcerative colitis
WHO	World Health Organization
